# Accurate strain sensing based on super-mode interference in strongly coupled multi-core optical fibres

**DOI:** 10.1038/s41598-017-04902-3

**Published:** 2017-06-30

**Authors:** Joel Villatoro, Oskar Arrizabalaga, Gaizka Durana, Idurre Sáez de Ocáriz, Enrique Antonio-Lopez, Joseba Zubia, Axel Schülzgen, Rodrigo Amezcua-Correa

**Affiliations:** 1 Department of Communications Engineering, Escuela de Ingeniería de Bilbao, University of the Basque Country (UPV/EHU), Alda. Urquijo s/n, E-48013 Bilbao, Spain; 2 IKERBASQUE—Basque Foundation for Science, E-48011 Bilbao, Spain; 3 Fundación Centro de Tecnologías Aeronáuticas (CTA), Miñano, Spain; 4 CREOL, The College of Optics & Photonics, University of Central Florida, P.O. Box 162700, Orlando, Florida 32816-2700 USA

## Abstract

We report on the use of a multi-core fibre (MCF) comprising strongly-coupled cores for accurate strain sensing. Our MCF is designed to mode match a standard single mode optical fibre. This allows us to fabricate simple MCF interferometers whose interrogation is carried out with light sources, detectors and fibre components readily available from the optical communications tool box. Our MCF interferometers were used for sensing strain. The sensor calibration was carried out in a high-fidelity aerospace test laboratory. In addition, a packaged MCF interferometer was transferred into field trials to validate its performance under deployment conditions, specifically the sensors were installed in a historical iron bridge. Our results suggest that the MCF strain sensors here proposed are likely to reach the readiness level to compete with other mature sensor technologies, hence to find commercial application. An important advantage of our MCF interferometers is their capability to operate at very high temperatures.

## Introduction

Optical fibre sensing is a viable technology to monitor strain, an important physical parameter in many application fields. Basically, fibre optic strain sensing consists of monitoring changes of phase, intensity, or wavelength of the guided light as the optical fibre is subjected to strain. There are multiple alternatives to devise fibre optic strain sensors, however, they lead to two main categories which are distributed and discrete (or point) sensors. The former require sophisticated interrogation systems and are suitable to monitor strain over long distances, on the order of tens of kilometres^1, 2^. On the other hand, point strain sensors are simpler and are used in applications that require the monitoring of strain at specific locations^3, 4^.

Currently, fibre Bragg grating (FBG) is the most prominent optical technology applied in high performance point strain sensors^5–7^. FBG strain sensors are widely used to monitor the integrity and health of civil and other critical infrastructures^8, 9^. Some drawbacks of FBG strain sensors include the need of expensive, picometre-resolution interrogators and their degradation at temperatures above a few hundred degrees Celsius. To overcome these limitations several research groups around the world are placing emphasis on the use of speciality optical fibres (SOFs). So far, diverse strain sensors based on SOFs have been proposed, including for example, photonic crystal fibres (PCFs)^10–13^, multi-core fibres with isolated cores^14–17^, and plastic optical fibres (POFs)^18–20^, amongst others. However, the lack of SOF components such as splitters, circulators, switches, etc., and the incompatibility of most SOFs with telecommunications optical fibres make SOF strain sensors complex and impractical. For these reasons the majority of strain sensors based on SOFs reported until now^10–20^ have been validated and tested only in conventional laboratory environments and most of their potential remains untouched.

Here, we report on an approach to build compact, simple and accurate fibre optic strain sensors based on particular SOFs, namely multi-core fibres (MCFs) with strongly coupled cores. Our MCF is designed to operate at optical telecommunication wavelengths and to support only two supermodes which are made to interfere in a highly predictable manner^21–24^. To fabricate our interferometric sensors, a short segment of MCF is inserted between two single mode fibres (SMF) by means of a conventional fusion splicing process. In the proposed architecture, strain causes drastic changes to the interfering supermodes, and consequently, easy-to-detect changes in the interference pattern^21–24^. We calibrated our sensors in a fatigue test bench in a high-fidelity aerospace test laboratory. In addition, some sensors were packaged and deployed for strain monitoring in a crossbeam of the *Vizcaya Bridge* (a UNESCO World Heritage Site) during a period of three months. In all the experiments our sensors were compared with commercial strain gauges and FBG sensors. Our results suggest that the strain sensors here proposed can compete in performance with such devices. An important advantage of our MCF interferometers is their ability to operate at elevated temperatures, up to 1000 °C^21^.

### Sensor fabrication and operation mechanism

**Figure 1:**
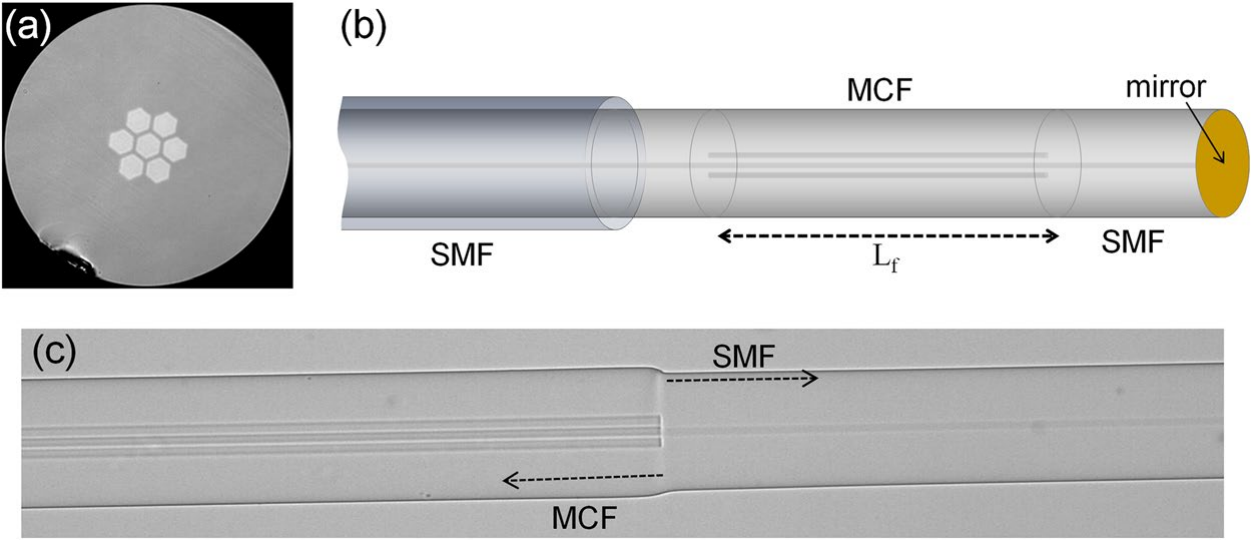
(**a**) Micrograph of the cross section of the MCF used to build strain sensors. (**b**) Sketch of a MCF interferometer. L_f_ is the length of the MCF. (**c**) Micrograph of one the MCF-SMF junctions.

The MCF used to fabricate the strain sensors consists of a core in the centre of the fibre surrounded by six identical cores, each with a diameter of 9.2 μm (Fig. 1a). All the cores of the MCF are made of germanium doped silica glass and are embedded in a pure silica cladding. The numerical aperture (NA), at 1550 nm, of the cores is 0.14 matching that of a conventional SMF. The distance between adjacent cores is 11 μm. The MCF was fabricated by a well-established stack and draw method^25^ at the University of Central Florida (Orlando, USA).

The structure of our devices is shown in Fig. 1b; it consists of a segment of MCF fusion spliced to two segments of SMF; one of them has a mirror at the end. To fabricate such a structure we used a specialty fibre fusion splicer (Fujikura 100 P+, Tokyo, Japan). The default splicing programmes installed in such a machine were used to join the SMF and the MCF. Under these conditions, the core of the SMF and the central core of the MCF get precisely aligned, and the optical fibres get permanently joined together; see Fig. 1c. As a result, the splice loss is minimal; on the order of 0.1 dB. In addition to that, the SMF-MCF junction has high tensile strength. Therefore, robust sensors with minimal insertion loss can be fabricated in a matter of minutes.

The interrogation of the device depicted in Fig. 1b simply entails a low-power broadband light source, a conventional fibre optic circulator or coupler, and a spectrum analyser. Unlike the interrogation of FBGs, the analysis and processing of interference patterns do not required high-resolution spectrum analysers. It has been theoretically and experimentally demonstrated that it is possible to detect picometer shifts of an interference pattern with low-resolution spectrometers by means of the discrete Fourier transform^26^. As the cost of nm-resolution spectrometers is low and their size is small, thus, it is feasible to implement low-cost, miniature, and portable interrogation systems for our MCF sensors.

**Figure 2:**
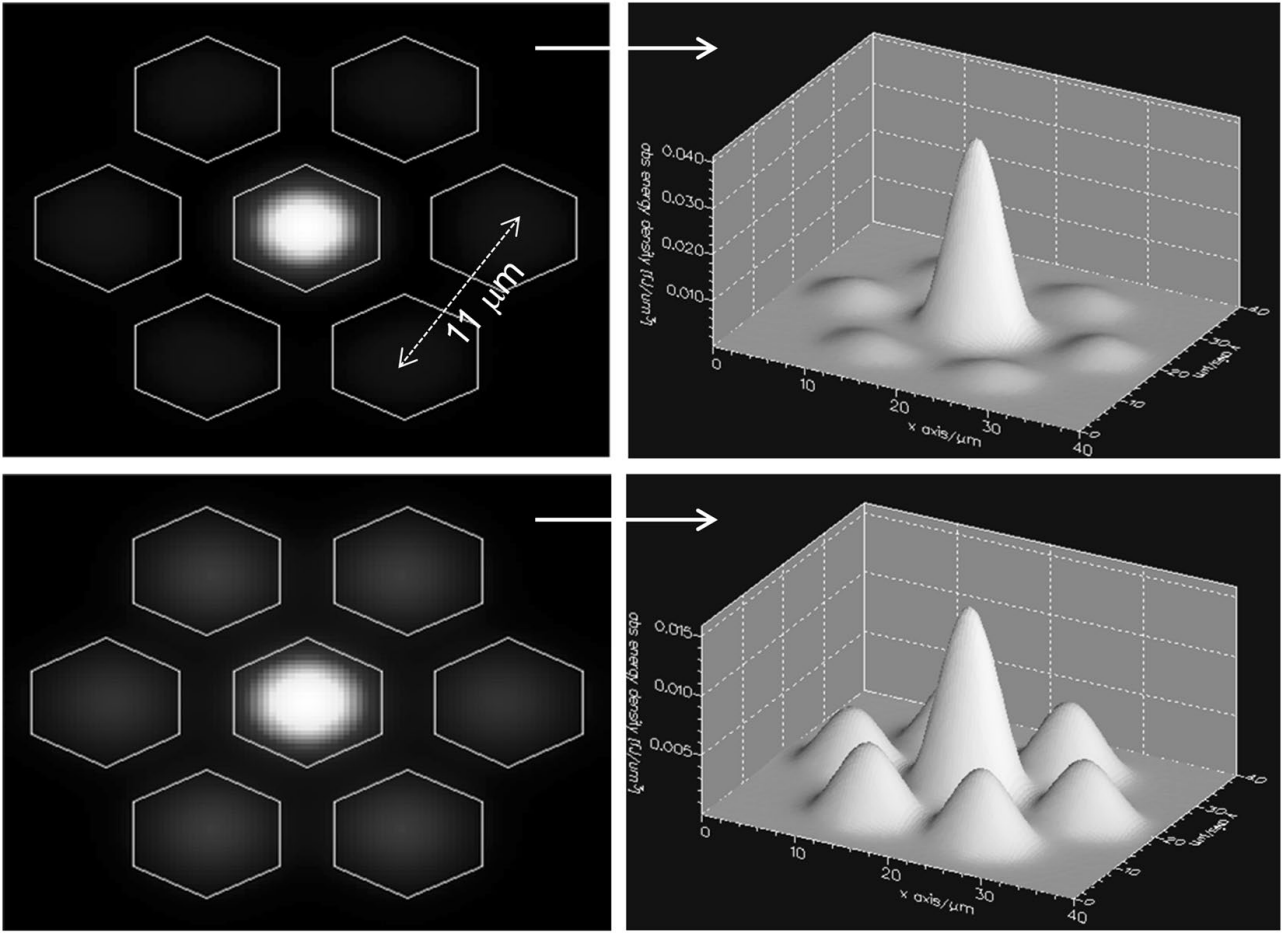
Simulated 2D and 3D mode profiles of the two supermodes excited in the MCF shown in Fig. 1(a).

In our devices the MCF plays a central role. The guided modes of a MCF with strongly coupled cores can be considered as the superposition of isolated *LP* modes supported by each core. For this reason, they are called supermodes^27, 28^. The design of the MCF along with the excitation conditions determine which and how many supermodes propagate and interfere in the MCF segment. Note that the SMF-MCF-SMF structure shown in Fig. 1b has axial symmetry and that the MCF is excited with the *LP*_01_ mode of a SMF. This results in exclusive excitation of two circularly symmetric supermodes with non-zero intensity in the central core of the MCF. The 2D and 3D mode profiles of the two supermodes that are excited in our MCF were simulated with commercial software (FimmWave by PhotonDesign, Oxford, UK). The results are shown in Fig. 2. For the simulations all the cores were considered to be identical with a diameter of 9.2 μm.

The two supermodes excited in the MCF have different effective refractive indices that can be termed as *n*_1_ and *n*_2_. Thus, the accumulated phase difference of the two supermodes when they pass twice the length L_f_ of the MCF is Δϕ = 4πL_f_Δ*n*/λ, where λ is the wavelength of the optical source and Δ*n* = *n*_1_ − *n*_2_. The reflection intensity of our device can be expressed as

1 $${I}_{{\rm{R}}}={I}_{1}+{I}_{2}+2\sqrt{{I}_{1}{I}_{2}}\,\cos ({\rm{\Delta }}\varphi ).$$

In Eq. (1) *I*_1_ and *I*_2_ are, respectively, the intensities of the supermode 1 and supermode 2. Thus, if different wavelengths are launched to the structure depicted in Fig. 1b and the reflected light is analysed with a spectrometer, a series of maxima and minima (interference pattern) is expected. It can be noted that when Δϕ = 2 *m*π, with *m* an integer, *I*_R_ will reach maximum values. Thus, the peaks of the interference pattern will appear at wavelengths that satisfy the expression

2 $${{\rm{\lambda }}}_{m}=(2{\rm{\Delta }}n{{\rm{L}}}_{{\rm{f}}})/m.$$

Now, if the MCF undergoes a minute elongation, δL_f_ (≪ *L*_*f*_), the interference pattern will shift. To determine the corresponding shift in wavelength (Δλ_*m*_) we can differentiate Eq. (2) with respect to L_f_ and obtain

3 $${{\rm{\Delta }}{\rm{\lambda }}}_{m}=[({L}_{{\rm{f}}}/{\rm{\Delta }}n)\partial ({\rm{\Delta }}n)/\partial {{\rm{L}}}_{{\rm{f}}}+1]{{\rm{\varepsilon }}{\rm{\lambda }}}_{m}.$$

In Equation (3), ε = δL_f_/L_f_, and according to ref. 29, ∂(Δn)/∂L_f_ ≈ −[*γ*/(2L_f_)]$({n}_{1}^{3}-{n}_{2}^{3})$ where γ is a constant that depends on the strain-optic coefficients and the Poisson ratio of the MCF. Equation (3) shows that Δλ_*m*_ (or shift of the interference pattern) depends linearly on the applied strain, is larger at longer wavelengths, and has a weak dependence on L_f_. This means that the length of MCF has not strong influence on the strain sensitivity of our devices.

**Figure 3:**
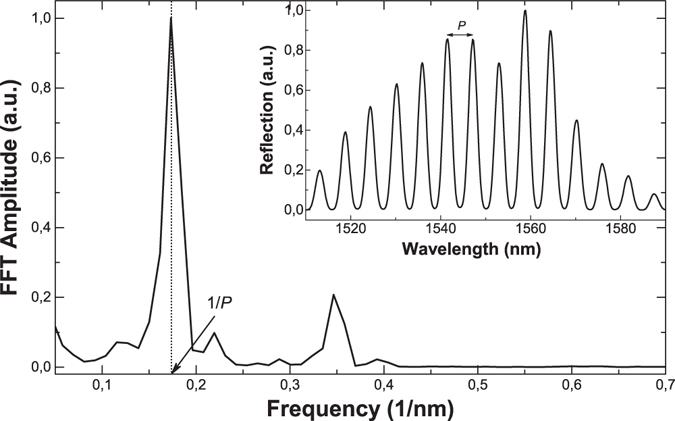
Amplitude of the FFT as a function of frequency of the interference pattern shown in the inset. The latter was observed in a device fabricated with L_f_ = 30 cm of MCF. *P* is the period of the interference pattern.

It is important to point out that Eqs (1) to (3) are valid when only two supermodes participate in the interference. To verify that this condition is fulfilled in our devices, we fabricated several interferometers with different lengths of MCF. To interrogate them we launched light from a superluminescent diode with peak emission at 1550 nm through a fibre optic circulator. The reflected spectrum was analysed with a miniature spectrometer (I-MON512-USB, from Ibsen Photonics, Farum, Denmark). The inset in Fig. 3 shows the reflected spectrum of a device built with 30 cm of MCF. Note the well defined series of maxima and minima. The minima close to 0 indicates almost perfect destructive interference or that the amplitudes of the excited supermodes are almost identical. Figure 3 also shows the normalized amplitude of the fast Fourier transform (FFT) of the spectrum shown in the inset as a function of frequency. Note that the FFT displays only one dominant peak at 1/*P*, where *P* is the period of the interference pattern. Figure 3 confirms that in our devices only two modes participate in the interference. This is possible since in a coupled-core MCF one has great control over the supermodes, their excitation and their interference through the coupling conditions and the MCF design. The latter includes the cores’ refractive indices and diameters, the number of cores and their separation^22^. Therefore, the proposed MCF interferometers can be superior for sensing applications compared to previously reported multimode fibre interferometers where the properties of the mode interference cannot be predicted as it is difficult to control the type and number of modes that participate in the interference^29–33^.

## Results and Discussion

**Figure 4:**
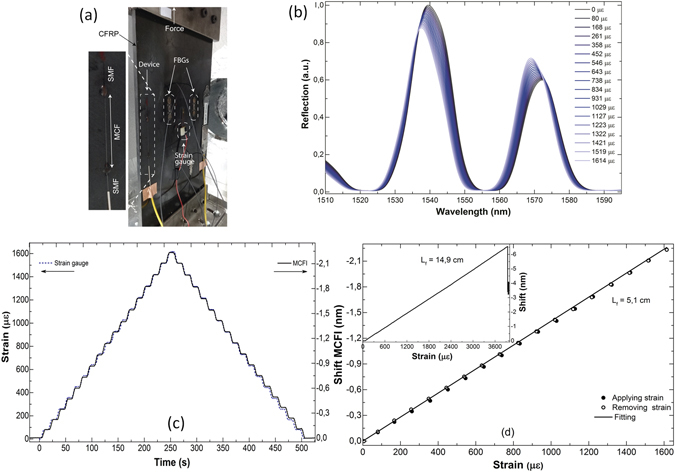
(**a**) Photograph of the specimen with several sensors bonded to its surface. (**b**) Interference patterns observed at different strain values. (**c**) Strain to the specimen measured with a strain gauge as a function of time and the corresponding shift of the interference pattern. (**d**) Calibration curve. In all cases L_f_ was 5,1 cm. The inset graph shows the strain versus shift observed in a 14, 9 cm long MCF interferometer.

The calibration of our devices was carried out at constant temperature (25 °C) in a fatigue test bench of the *Aerospace Technology Centre* (Miñano, Spain). A pre-strained interferometer built with 5.1 cm of MCF was bonded with epoxy resin on a specimen of carbon fibre reinforced polymer (CFRP) with dimensions of 41 × 18 × 0.62 cm. A linear strain gauge (model LY43-3/350 from HBM, Darmstadt, Germany) and two FBG strain sensors (model os3150 from MicronOptics, Atlanta, USA) were also bonded on the CFRP specimen for calibration purposes (see Fig. 4a). The specimen was subjected to axial tensile stress by means of a computer-controlled hydraulic system (servocylinder hydraulic, model CIL125/80/80 × 500, from Glual Hidráulica S.L., Gipuzkoa, Spain) compliant with the ISO6020/2 standard. The experiments were carried out several times on three different days.

Figure 4b shows the evolution of the interference patterns for different stresses applied to the specimen. Figure 4c shows the strain measured with the strain gauge and the corresponding shifts of the interference pattern of our MCF sensor. The interference pattern shift was calculated as the average of the individual shifts of the two broad interference peaks shown in Fig. 4a. The individual shifts of the peaks shown in Fig. 4b were calculated by means of the centre of gravity algorithm which is capable of detecting pm shifts even when the peaks are broad^34^. It should be noted that the high spectral resolution of our Ibsen spectrometer was not exploited. The calibration curve is shown in Fig. 4d. The calibration curve gave us the relationship between strain (in με) and wavelength shift (in pm) as ε = Δλ_*m*_/(−0.0014 pm/με). This means that the sensitivity of our 5.1 cm-long device is 1.4 pm/με. It should also be noted that the MCF sensor exhibits negligible hysteresis

We also investigated the influence of the length of MCF on the strain sensitivity of the sensors and the maximum strain that the MCF interferometers could measure. To do so, we fabricated an interferometer with 14,9 cm of MCF; it was subjected to axial strain in a computer-controlled material testing system (Model 5980, Instron, Norwood, MA, USA) until it was broken. The shift of the interference pattern as a function of the applied strain is shown in the inset of Fig. 4d. The strain sensitivity of the 14, 9 cm-long sample was found to be ~1.7 pm/με which is ~40% higher than that of conventional FBG strain sensors^5, 6^. It is important to point out that the maximum strain that can be measured with our devices is determined by the quality of the splices. In our case, the MCF-SMF junctions are as robust as SMF-SMF splices. The maximum strain that our 14, 9 cm-long MCF interferometer withstood was ca 3800 με which is sufficient for most real-world strain sensing applications.

The results shown in Fig. 4 demonstrate that the novel, prototype MCF strain sensor reported here compares well with high performance strain gauges in regard to accuracy and resolution. Thus, wherever traditional strain gauges cannot be applied because of specific requirements such as need of embeddable sensors, available space, remote monitoring, or temperature of operation, MCF sensors can provide a suitable alternative.

**Figure 5:**
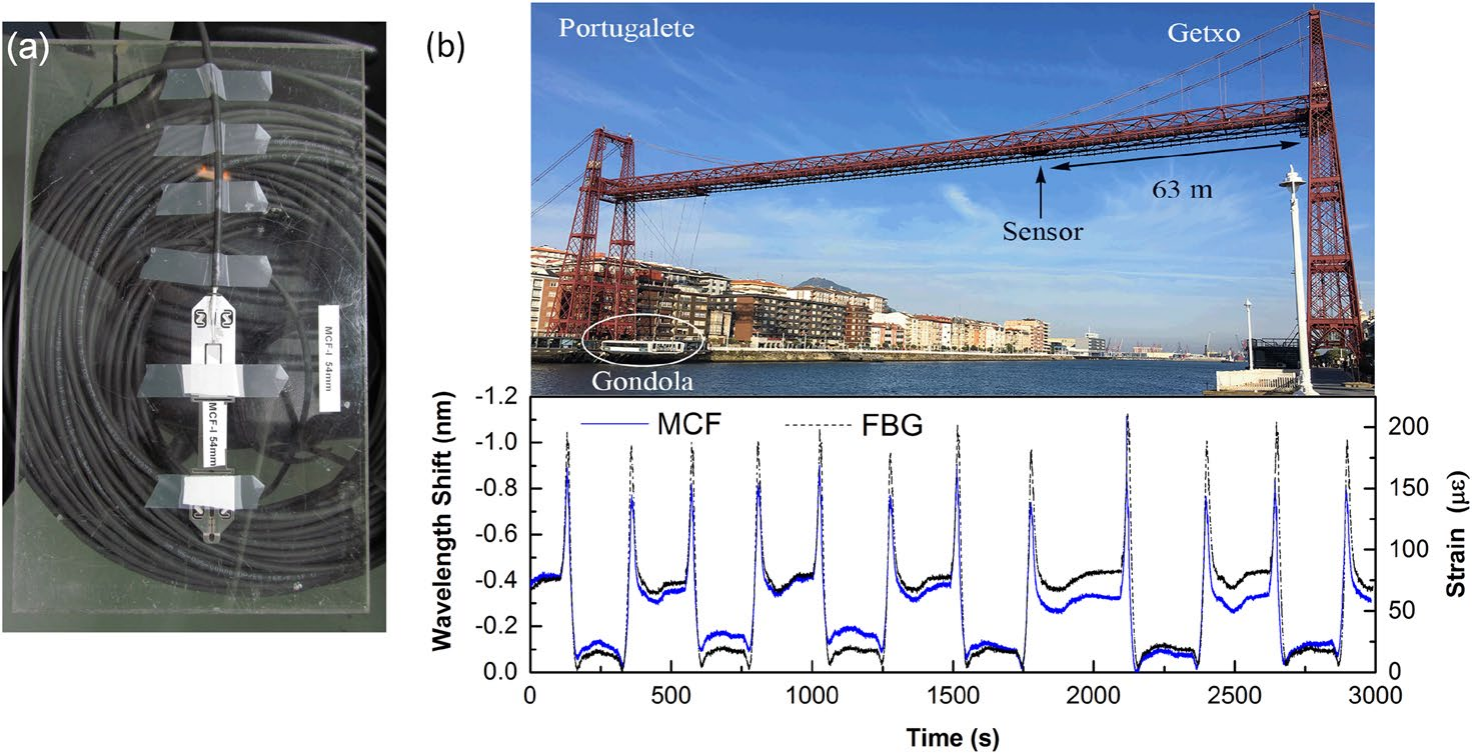
(**a**) Photograph of a packaged MCF sensor. The black cable is the 100 m long patchcord. (**b**) Photograph of the *Vizcaya Bridge* highlighting the position of the sensor and the hanging gondola. The bottom graph shows the shift of the interference pattern of our MCF sensor and the strain measured by an FBG sensor at the same point of the crossbeam of the bridge while the gondola moved from Portugalete to Getxo and back.

The capability of our interferometer to operate in a field environment was also investigated. For such purpose, we fabricated an interferometer with a 5.4 cm of MCF and packaged it with a rugged strain gage made of 302 stainless steel (model os3155, provided by MicronOptics, Atlanta, USA). Such a gage is especially designed to transfer strain from metallic structures to an optical fibre. The MCF sensor had a 100 m long patchcord made of G657. A2 cable designed for outdoor use. A photograph of the packaged MCF sensor is shown in Fig. 5a. To reflect the guided light we cleaved the distal SMF and protected it with a segment of capillary tube. The MCF interferometer was spot welded to a crossbeam of the 120 year-old *Vizcaya Bridge*, declared a World Heritage Site by UNESCO in 2006. A temperature-compensated FBG strain sensor (os3155 from MicronOptics) was also welded to the crossbeam, close to our MCF sensor. The FBG sensor was used as reference and also for temperature compensation of our MCF sensor.

The structure of the *Vizcaya Bridge* consists of two 160 m-long crossbeams supported by four 61 m-height towers (see Fig. 5b). All the structure is made of iron. All the pieces that form the bridge are joined with rivets and cables. The particular feature of the bridge is that a gondola hangs from the horizontal crossbeams. In about 80 seconds the gondola can transport up to 200 passengers and six cars from one town (Getxo) to another one (Portugalete) which are separated by a river (see Fig. 5b). It is important to point out that under normal conditions the gondola makes 2140 trips per week, or more than 8500 trips per month. Therefore, to preserve the integrity of such a historical bridge it is crucial to monitor the strain on critical points of the crossbeams, cables and towers over time or when the gondola moves. Thus, in a pilot test we welded our packaged MCF interferometer and the commercial FBG strain sensor at a point located 63 m from the towers in the Getxo side (see Fig. 5b). The FBG strain sensor was interrogated with a sm125 Interrogator (from MicronOptics) and our MCF interferometer with a miniature spectrometer (mentioned above) that operates in the 1510–1590 nm wavelength range. The data were collected on arbitrary days during three months. To compensate temperature effects on our MCF interferometer we used the temperature sensor of the FBG strain sensor. According to ref. 21 the thermal sensitivity of our MCF interferometer was considered as 30 pm/°C.

The graph of Fig. 5b shows the typical shift of the interference pattern observed in our MCF interferometer and the strain measured with the FBG sensor as a function of time when the gondola moved from Getxo to Portugalete and back to Getxo. As expected the shift of our MCF interferometer (or strain in the FBG sensor) was nearly 0 nm (or 0 με) when the gondola was further away from the sensor (in Portugalete) and maximum, see the peaks in the bottom graph of Fig. 5b, when the gondola passed beneath the sensor. The regions between two consecutive maxima were observed either when the gondola was in Getxo or in Portugalete. Due to the closer proximity of the sensors to the Getxo side they measured higher strain when the gondola was at the Getxo side. Note that our device provides essentially the same information than a well-calibrated commercial FBG strain sensor. This clearly demonstrates the potential of MCF sensors to work in real-world environments.

## Conclusions

In this paper, we have reported on strain sensing based on interference of two supermodes in a precisely designed MCF with strongly coupled cores. The interferometers reported here consist of a short segment of MCF, inserted, via standard fusion splicing, between two single mode fibres. The fabrication of the SMF-MCF-SMF structure is fast, reproducible and inexpensive as it is carried out with hardware widely used in the telecommunications industry, and conventional fibre splicing routines.

The calibration of our strain sensors was performed in a high-fidelity test laboratory following procedures of the aerospace industry. We have shown that the proposed devices are as accurate and sensitive as strain gauges or FBG sensors. In addition, we packaged some MCF interferometers with strain gages used to package commercial FBG sensors and tested them in a real-world environment, specifically, at a historic iron bridge located close to Bilbao, Spain. We found that in this field trial our MCF sensor provides the same information as a commercial FBG strain sensor. To the authors’ best knowledge, this is the first time a sensor based on special MCF is tested, validated and compared with well-established fibre sensors in a field environment which represents a great leap towards commercial applications.

Finally, we would like to point out that the multiplexing of our MCF interferometers is feasible, although it is not demonstrated here. A straightforward method to interrogate N MCF sensors is the application of a 1xN fibre optic switch. The switching speed of such devices is on the order of milliseconds. Therefore, it is possible to implement networks of MCF sensors. Combining their high performance, as shown by the measurements performed at the Vizcaya bridge, with their small packaging, symplified interrogation and the possibility of operation at elevated temperatures, MCF sensors could open new avenues and markets for fibre optic point sensors.

## References

[1] Bao X, Chen L (2012). Recent progress in distributed fiber optic sensors. Sensors.

[2] Motil A, Bergman A, Tur M (2016). State of the art of Brillouin fiber-optic distributed sensing. Opt. Laser. Technol..

[3] Kersey AD (1996). A review of recent developments in fiber optic sensor technology. Opt. Fiber Technol..

[4] Grattan KTV, Sun T (2000). Fiber optic sensor technology: an overview. Sensor Actuat. A-Phys.

[5] Hill KO, Meltz G (1997). Fiber Bragg grating technology fundamentals and overview. J. Lightwave Technol..

[6] Rao YJ (1997). In-fibre Bragg grating sensors. Meas. Sci. Technol..

[7] Mihailov SJ (2012). Fiber Bragg grating sensors for harsh environments. Sensors.

[8] Majumder M, Gangopadhyay TK, Chakraborty AK, Dasgupta K, Bhattacharya DK (2008). Fibre Bragg gratings in structural health monitoring -Present status and applications. Sensor Actuat. A-Phys.

[9] López-Higuera JM, Cobo LR, Incera AQ, Cobo A (2011). Fiber optic sensors in structural health monitoring. J. Lightwave Technol..

[10] Villatoro, J., Finazzi, V., Minkovich, V. P., Pruneri, V., Badenes, G. Temperature-insensitive photonic crystal fiber interferometer for absolute strain sensing. *Appl*. *Phys*. *Lett*. **91**, art. num. 091109 (2007).

[11] Han YG (2009). Temperature-insensitive strain measurement using a birefringent interferometer based on a polarization-maintaining photonic crystal fiber. Appl. Phys. B..

[12] Qureshi KK, Liu ZY, Tam HY, Zia MF (2013). A strain sensor based on in-line fiber Mach–Zehnder interferometer in twin-core photonic crystal fiber. Opt. Commun..

[13] Naeem K, Kwon IB, Chung Y (2017). Multibeam interferometer using a photonic crystal fiber with two asymmetric cores for torsion, strain and temperature sensing. Sensors.

[14] Flockhart GMH (2003). Two-axis bend measurement with Bragg gratings in multicore optical fiber. Opt. Lett..

[15] Moore JP, Rogge MD (2012). Shape sensing using multi-core fiber optic cable and parametric curve solutions. Opt. Express.

[16] Barrera D, Gasulla I, Sales S (2015). Multipoint two-dimensional curvature optical fiber sensor based on a nontwisted homogeneous four-core fiber. J. Lightwave Technol..

[17] Li C (2016). All-fiber multipath Mach-Zehnder interferometer based on a four-core fiber for sensing applications. Sensor Actuat. A-Phys.

[18] Peters, K. Polymer optical fiber sensors—a review. *Smart Mater*. *Struct*. **20**, art. ID 013002 (2010).

[19] Bilro L, Alberto N, Pinto JL, Nogueira R (2012). Optical sensors based on plastic fibers. Sensors.

[20] Webb, D. J. Fibre Bragg grating sensors in polymer optical fibres. *Meas*. *Sci*. *Technol*. **26**, art ID 092004 (2015).

[21] Antonio-Lopez JE, Eznaveh ZS, LiKam WaP, Schülzgen A (2014). Amezcua-Correa, R. Multicore fiber sensor for high-temperature applications up to 1000 °C. Opt. Lett..

[22] Van Newkirk A, Antonio-Lopez JE, Salceda-Delgado G, Amezcua-Correa R, Schülzgen A (2014). Optimization of multicore fiber for high temperature sensing. Opt. Lett..

[23] Amezcua-Correa, R., Schülzgen, A., Antonio-Lopez, J. E. Multicore optical fiber apparatus, methods, and applications. US patent WO2015163963A2 (December 17, 2015).

[24] Salceda-Delgado G (2015). Compact fiber-optic curvature sensor based on super-mode interference in a seven-core fiber. Opt. Lett..

[25] Knight JC, Birks TA, Russell PJ, Atkin DM (1996). All-silica single-mode optical fiber with photonic crystal cladding. Opt. Lett..

[26] Misiakos K (2014). Broad-band Mach-Zehnder interferometers as high performance refractive index sensors: Theory and monolithic implementation. Opt. Express.

[27] Xia C, Bai N, Ozdur I, Zhou X, Li G (2011). Supermodes for optical transmission. Opt. Express.

[28] Xia C (2016). Supermodes in coupled multi-core waveguide structures. IEEE J. Sel. Top. Quantum. Electron..

[29] Kumar A, Goel NK, Varshney RK (2001). Studies on a few-mode fiber-optic strain sensor based on LP_01_–LP_02_ mode interference. J. Lightwave Technol..

[30] Wang Q, Farrell G (2006). All-fiber multimode-interference-based refractometer sensor: proposal and design. Opt. Lett..

[31] Li, E., Wang, X., Zhang, C. Fiber-optic temperature sensor based on interference of selective higher-order modes. *Appl*. *Phys*. *Lett*. **89**, art. 091119 (2006).

[32] Salceda-Delgado G, Monzon-Hernandez D, Martinez-Rios A, Cardenas-Sevilla GA, Villatoro J (2012). Optical microfiber mode interferometer for temperature-independent refractometric sensing. Opt. Lett..

[33] Villatoro J, Minkovich VP, Zubia J (2015). Photonic crystal fiber interferometric force sensor. IEEE Photon. Tech. Lett.

[34] Villatoro J, Antonio-Lopez JE, Schülzgen A, Amezcua-Correa R (2017). Miniature multicore optical fiber vibration sensor. Opt. Lett..

